# Impact of antiplatelet therapy on outcomes of sepsis: A systematic review and meta-analysis

**DOI:** 10.1371/journal.pone.0322293

**Published:** 2025-04-29

**Authors:** Xufang Wang, Huifei Zhou

**Affiliations:** Department of Critical Care Medicine, Huzhou Third Municipal Hospital, the Affiliated Hospital of Huzhou University, Huzhou, Zhejiang, China; Azienda Ospedaliero Universitaria Careggi, ITALY

## Abstract

**Objective:**

Antiplatelet therapy has been studied for its potential benefits in various cardiovascular conditions, but its role in sepsis remains less clear. This review aims to systematically analyse the available evidence on the effects of antiplatelet therapy in sepsis to assess its potential benefits and risks.

**Material and methods:**

The studies published until 01^st^ April 2024 from PubMed, Embase and Scopus databases were searched. Pooled effect sizes were reported as relative risks (RR) or weighted mean difference (WMD) with corresponding 95% confidence intervals (CI). Outcomes included mortality, length of intensive care unit (ICU) stay, hospital stay, and the risk of complications. The certainty of evidence was evaluated using GRADE.

**Results:**

Twenty-one studies were included. Antiplatelet therapy was associated with significantly lower risk of in-hospital mortality (RR 0.76, 95% CI: 0.67, 0.87), and mortality at one (RR 0.77, 95% CI: 0.66, 0.90) and three months (RR 0.77, 95% CI: 0.66, 0.90) follow up. Risk of complications was comparable in all patients (RR 1.01, 95% CI: 0.84, 1.21). ICU stay (in days) (WMD -0.23, 95% CI: -0.53, 0.07; N=7, I^2^=97.2%) and overall duration of hospital stay (in days) (WMD 0.63, 95% CI: -0.66, 1.92; N=6, I^2^=93.2%) was also statistically similar among patients who received and did not receive antiplatelet drugs. The certainty of evidence for the outcomes ranged from “low to very low”.

**Conclusion:**

Antiplatelet therapy appears safe and significantly lowers the risk of short-term mortality in septic patients. While antiplatelet therapy did not impact the duration of ICU or overall hospital stay, our findings underscore the potential of antiplatelet agents as a beneficial adjunctive therapy in sepsis management.

## Introduction

According to the Global Burden of Disease Study (2020), sepsis accounts for around one-fifth of all reported deaths worldwide, with around 49 million cases of sepsis and 11 million sepsis-related deaths worldwide [1]. Global sepsis incidence is documented to be higher among women than men (717 vs. 643 cases per 100, 000); however, sepsis related mortality is higher among men (164 vs. 134 per 100, 000 respectively) [2]. Furthermore, gross regional and economic disparities have been found, i.e., 85.0% of sepsis cases and 84.8% of related deaths worldwide occur in countries with low, low-middle, or middle sociodemographic indices, particularly in sub-Saharan Africa and South-East Asia [2]. It is also a predominant cause of hospitalisation in the intensive care units (ICUs), often culminating in multiple organ dysfunction and death [3], and is associated with significant economic burden [4]. A recent systematic review on the hospital related cost of sepsis found a wide disparity in sepsis related costs across different countries and ranged from 1300 to 100,000 USD per patient [5]. Numerous studies showed that inflammation and coagulation play a pivotal role in pathophysiology of sepsis [6–8]. Impaired coagulative function may lead to disseminated intravascular coagulation, resulting in the formation of intravascular microthrombi [9,10], and release of inflammatory factors due to the intricate interplay between platelets and endothelial cells, and platelet-neutrophil interactions [11,12]. Therefore, understanding potential benefits of antiplatelet agents is crucial to improve outcomes for patients with sepsis.

The existing evidence regarding the impact of antiplatelet therapy on sepsis outcomes remains inconclusive. Some studies indicate that antiplatelet drugs may improve survival rates and shorten hospital stays in sepsis patients; however, other research either finds no significant effect on key clinical outcomes or raises concerns about potential risks associated with their use in sepsis. [13–16]. For instance, while ticagrelor has been shown to reduce pneumonia risk in some contexts [17], its impact on sepsis outcomes remains controversial, as highlighted in the debates around the PLATO trial [18]. A prior systematic review by Ouyang et al, encompassing ten cohort studies, demonstrated a substantial reduction in mortality (nearly 18%) with the use of antiplatelet therapy [19]. An individual-patient data meta-analysis by Trauer et al, including data from fifteen hospital-based cohorts and a large insurance-based database, showed that the preventive use of aspirin before sepsis onset was linked to a 7% reduction in the risk of death [20].

The existing literature reflects a complex relationship between antiplatelet therapy and sepsis outcomes, with varying results across different studies. This inconsistency could be due to differences in study design, patient populations, or the types of antiplatelet agents used. This updated review is intended to contribute valuable insights to the evolving understanding of the role of antiplatelet therapy in influencing outcomes for individuals with sepsis, thereby, facilitating informed decision-making in clinical setting.

## Methods

The meta-analysis adhered to the PRISMA guidelines [21], and the study protocol was registered in PROSPERO (Registration number CRD42023486016) prior to the start of the review.

### Databases used and search strategy

Database search identified studies from PubMed, Embase, and Scopus using the following structured search strategy for each of these databases (S1 Table). Our search was limited to English language studies published until April 01, 2024. Manual search of references and reviews was also done.

### Criteria for inclusion and exclusion

*Inclusion criteria*: 1) Age ≥ 18 years or above) with confirmed sepsis based on established criteria; 2) Studies with an exposure group of patients who received antiplatelet drugs either at the time of sepsis diagnosis, during sepsis management, or maximum of 3 months before sepsis diagnosis; 3) Studies with a control group of patients who did not receive antiplatelet therapy either before, during, or after a sepsis diagnosis; 4) No history of drugs that could interfere with platelet functioning in the control group, i.e., patients with sepsis should not have received any medications that could interfere with platelet function, such as anticoagulants (e.g., warfarin, heparin), non-steroidal anti-inflammatory drugs (NSAIDs), and other drugs like selective serotonin reuptake inhibitors (SSRIs) that may alter platelet aggregation; 5) Studies reporting on at least one of the specified outcomes of interest: mortality (all-cause mortality rate reported within 12 months of follow up), length of ICU or hospital stay (duration that patients spend in the ICU and the total number of days the patient is hospitalized, from admission to discharge. These serve as an indicator of the overall burden and severity of illness as well as resource utilization), and the risk of complications (defined as any adverse events or conditions that arise during the course of sepsis management); 5) Studies that present the effect size, either as odds ratio (OR) or relative risk (RR) along with 95% confidence intervals (CI); 6) Observational studies such as cohort studies and case-control studies, as well as randomized controlled trials for inclusion; 7) English-language and peer-reviewed studies.

*Exclusion criteria*: 1) Case reports, letters, reviews, and conference abstracts; 2) Duplicate reports; 3) Studies with pregnant or lactating women; 4) Studies that do not clearly disclose treatment involving antiplatelet drugs, or whether the control group did not receive antiplatelet therapy; 5) Studies with incomplete or unclear data regarding relevant outcomes.

### Process of identifying eligible studies

#### Title and abstract screening.

After executing the search strategy, the initial list of studies was compiled after deduplication. Two independent reviewers conducted an initial screening of all identified studies based on their titles and abstracts. The primary focus during this stage was to exclude studies that were clearly irrelevant to the review’s inclusion criteria (e.g., studies not involving sepsis or antiplatelet therapy).

#### Full-text screening.

Studies that appeared potentially relevant based on the title and abstract were retrieved in full text and assessed for eligibility by the same two independent reviewers. The reviewers evaluated the studies against the predefined inclusion and exclusion criteria.

#### Conflict resolution.

If there were any discrepancies or disagreements between the two reviewers regarding the inclusion or exclusion of a study, the conflicting decisions were documented, and both reviewers discussed the differences to understand the rationale behind each decision. Studies were included in the review only after both reviewers reached consensus. This process ensured that the selection of eligible studies was unbiased and consistent with the review’s objectives.

#### Documentation and quality control.

All decisions, including reasons for exclusion, were documented to maintain transparency. Throughout the screening process, regular meetings were held to discuss any ongoing issues, refine the inclusion/exclusion criteria if necessary, and ensure consistency in decision-making.

### Data extraction

A standardized data extraction form was used independently by two authors. The data on key variables were extracted using a standardized data extraction form. These included study details such as the title, authors, year of publication, study design and sample size; subject characteristics such as age and gender distribution as well as key comorbidities; characteristics of antiplatelet administration such as the type of antiplatelet therapy used and the timing of therapy in relation to sepsis diagnosis (before, during, or after diagnosis); confirmation of no antiplatelet or platelet-affecting drug use in the comparison group; and outcomes, i.e., mortality, length of ICU stay and hospital stay as well as risk of complications. Any discrepancies were resolved by discussions.

### Risk of bias assessment and statistical analysis

Risk of bias was evaluated using the Newcastle-Ottawa Scale (NOS) [22]. The scale has been developed to assess the risk of bias by evaluating three domains: Selection, Comparability, and Outcome/Exposure assessment. (A) Selection (maximum score of 4): This domain assessed the representativeness of the exposed cohort (or case group in case-control study), the selection of the non-exposed cohort (or control group), ascertainment of exposure, and the demonstration that the outcome of interest was not present at the start of the study; (B) Comparability (maximum score of 2): This domain focused on whether the study design or analysis adequately controlled for confounding variables. Studies were awarded points if they accounted for key confounders in their design or analysis; (C) Outcome/Exposure (maximum score of 3): For cohort studies, this domain evaluated the methods used to assess outcomes and if there was significant loss to follow-up. For case-control study, this domain assessed the ascertainment of exposure and the reliability of the exposure assessment.

Pooled effect sizes were reported as relative risks (RR) for categorical outcomes and weighted mean difference (WMD) for continuous outcomes along with corresponding 95% CI. We converted ORs to RRs to maintain consistency across studies and enhance comparability of effect estimates. The conversion was performed using the well-established method by Zhang & Yu (1998), using the formula: RR=OR/[(1−P0)+(P0×OR)]; where P₀ represents the baseline risk (control event rate)[23]. A decision was made to use random-effects model for all analyses. This decision was based on the assumption that the studies included in the meta-analysis were not identical in terms of design, population, settings and exposure characteristics. The random-effects model accounts for both within-study and between-study variability, acknowledging that the effect size may vary across studies due to different populations, settings, or exposures/interventions. Further, the random-effects model provides a more conservative estimate by widening confidence intervals, making the results more generalizable to a broader range of clinical settings. Publication bias was assessed using Egger’s test and funnel plots [24]. A funnel plot visually assesses publication bias by plotting the effect sizes of studies against their standard errors. Egger’s test was performed to statistically evaluate the asymmetry of the funnel plot, where a significant p-value (p < 0.05) indicated potential publication bias. A subgroup analysis was performed based on the specifications of antiplatelet drug, timing of administration (prior to or after the diagnosis of sepsis) and sample size of the studies (<500 and ≥500). Significance was set at P<0.05. The Grading of Recommendations Assessment, Development and Evaluation (GRADE) framework was employed to determine the overall certainty of the evidence for each outcome [25].

## Results

**Fig 1:**
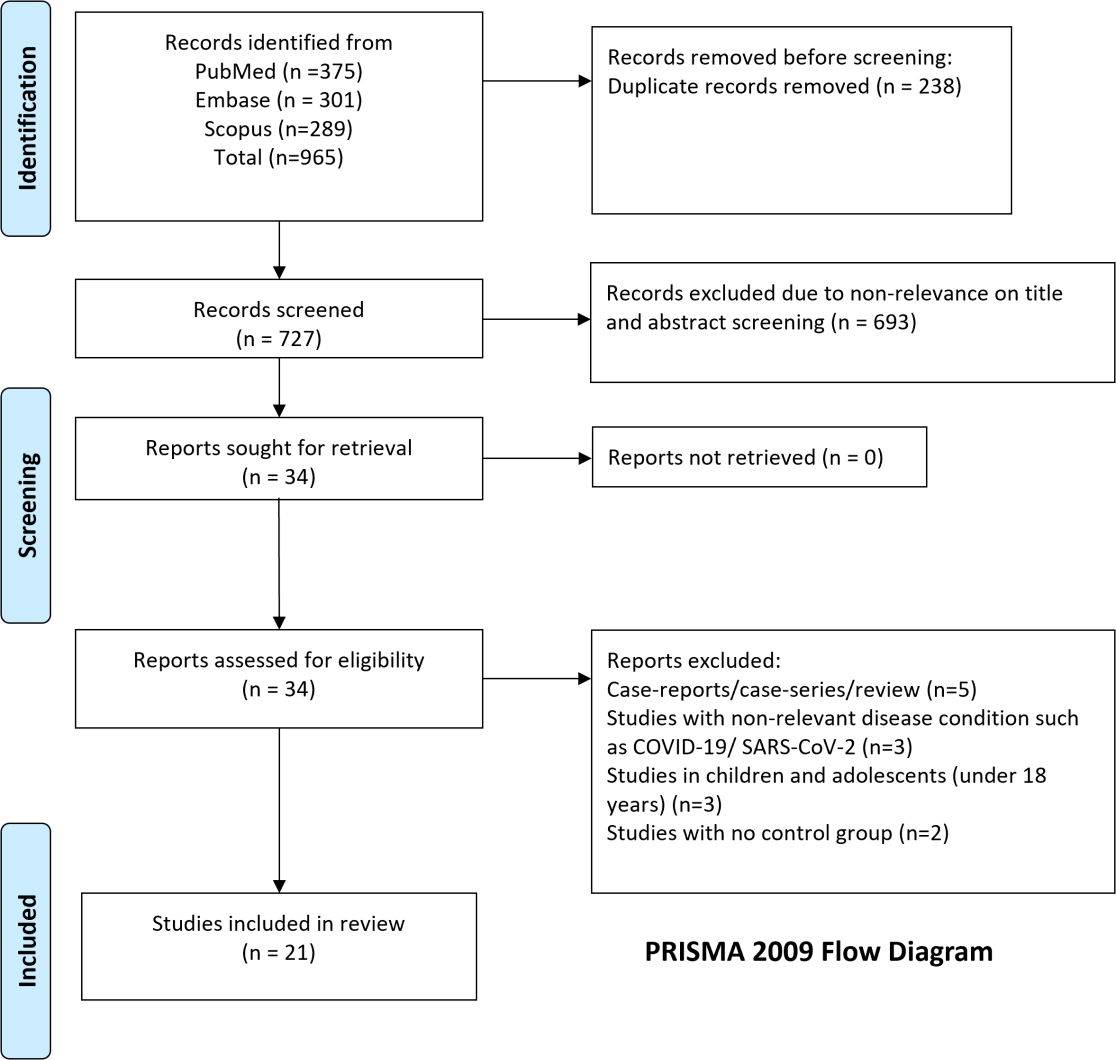
Selection process of studies included in the review.

A total of 965 studies were retrieved (Fig 1). Following the removal of 238 duplicate papers, 727 unique studies remained. In the initial screening, based on titles and abstracts, 693 studies were eliminated as ineligible (S2 Table). During the title and abstract screening, we included studies that focused on adult patients (age ≥ 18 years) with confirmed sepsis and investigated the use of antiplatelet therapy, while excluding studies involving paediatric populations and non-sepsis conditions. Additional 13 studies were excluded after full-text evaluation, leaving 21 eligible studies [13–16,26–42] (Table 1, S3 Table). For the full-text evaluation, we based our selection on the inclusion and exclusion criteria already described in the methods section. Specifically, we included studies that provided clear comparisons between patients receiving antiplatelet therapy and those not receiving it and reported effect sizes with confidence intervals for the outcomes of interest. We excluded studies that lacked sufficient outcome data or had overlapping or duplicated information.

**Table 1:** Characteristics of the included studies.

Author first name, year	Location	Study nature	Age and gender	Characteristics of antiplatelet administration	Sample size	Newcastle- Ottawa score
Lu (2023) [26]	China	Retrospective cohort	Mean age of around 65 years Male (56%)	Timing of antiplatelet administration- before onset of sepsis Antiplatelet drug used- Aspirin	2090 (with antiplatelet treatment- 621; without antiplatelet treatment- 1469)	7
Kim (2023) [27]	Republic of Korea	Retrospective cohort	Mean age of around 69 years Male (51%)	Timing of antiplatelet administration- before onset of sepsis Antiplatelet drug used- Aspirin/NSAIDs	241 (with antiplatelet treatment- 76; without antiplatelet treatment- 165)	7
Chen (2023) [28]	China	Retrospective cohort	Median age of around 69 years Male (56%)	Timing of antiplatelet administration- after onset of sepsis Antiplatelet drug used- Aspirin	7694 (with antiplatelet treatment- 3847; without antiplatelet treatment- 3847)	8
Wang_A (2023) [29]*	China	Retrospective cohort	Median age of around 68 years Male (57%)	Timing of antiplatelet administration- after onset of sepsis Antiplatelet drug used- Aspirin	15397 (with antiplatelet treatment- 2261; without antiplatelet treatment- 13136)	8
Wang_B (2023) [29]*	China	Retrospective cohort	Median age of around 69 years Male (57%)	Timing of antiplatelet administration- after onset of sepsis Antiplatelet drug used- Aspirin	3759 (with antiplatelet treatment- 727; without antiplatelet treatment- 3052)	8
Kobayashi (2022) [30]	Japan	Retrospective cohort	Mean age of around 70 years Male (>60%)	Timing of antiplatelet administration- before onset of sepsis Antiplatelet drug used- Not mentioned.	1184 (with antiplatelet treatment- 175; without antiplatelet treatment- 1009)	8
Hsu (2022) [13]	Taiwan	Retrospective cohort	Mean age of around 67 years Male (58%)	Timing of antiplatelet administration- prior to onset of sepsis Antiplatelet drug used- Aspirin	51857 (with antiplatelet treatment- 12776; without antiplatelet treatment- 39081)	8
Jain (2022) [31]	USA	Retrospective cohort	Median age of around 63 years Male (54%)	Timing of antiplatelet administration- prior to onset of sepsis Antiplatelet drug used- Clopidogrel	44619 (with antiplatelet treatment- 42523; without antiplatelet treatment- 2096)	7
Lavie (2022) [14]	Israel	Retrospective cohort	Median age of around 77 years Male (52%)	Timing of antiplatelet administration- prior to onset of sepsis Antiplatelet drug used- Aspirin	1066 (with antiplatelet treatment- 533; without antiplatelet treatment- 533)	8
Rögnvaldsson (2022) [32]	Iceland	Retrospective cohort	Median age of around 70 years Male (54%)	Timing of antiplatelet administration- before onset of sepsis Antiplatelet drug used- aspirin	815 (with antiplatelet treatment- 128; without antiplatelet treatment- 687)	8
Hsu (2018) [33]	USA	Retrospective cohort	Mean age of around 65 years Male (45%)	Timing of antiplatelet administration- prior to onset of sepsis Antiplatelet drug used- Aspirin	29690 (with antiplatelet treatment- 12869; without antiplatelet treatment- 16821)	7
Sahin (2018) [34]	Turkey	Retrospective cohort	Mean age of around 63 years Male (59%)	Timing of antiplatelet administration- before onset of sepsis Antiplatelet drug used- aspirin	64 (with antiplatelet treatment- 32; without antiplatelet treatment- 32)	6
Al Harbi (2016) [15]	Kingdom of Saudi Arabia	Nested case-control	Mean age of around 55 years Male (66%)	Timing of antiplatelet administration- after onset of sepsis Antiplatelet drug used- aspirin	763 (with antiplatelet treatment- 154; without antiplatelet treatment- 609)	7
Wiewel (2016) [16]	Netherlands	Prospective cohort	Mean age of around 62 years Male (60%)	Timing of antiplatelet administration- before onset of sepsis Antiplatelet drug used- aspirin, Clopidogrel, Dipyridamole, Prasugrel	972 (with antiplatelet treatment- 267; without antiplatelet treatment- 705)	8
Osthoff (2016) [35]	Switzerland	Retrospective cohort	Mean age of around 60 years Male (65%)	Timing of antiplatelet administration- before onset of sepsis Antiplatelet drug used- aspirin	689 (with antiplatelet treatment- 157; without antiplatelet treatment- 532)	7
Tsai (2015) [36]	Taiwan	Retrospective cohort	Mean age of around 67 years Male (54%)	Timing of antiplatelet administration- prior to onset of sepsis Antiplatelet drug used- aspirin/clopidogrel/ticlopidine	683421 (with antiplatelet treatment- 117447; without antiplatelet treatment- 565974)	7
Campbell (2015) [37]	United Kingdom	Retrospective cohort	Median age of around 70 years Male (50%)	Timing of antiplatelet administration- prior to onset of sepsis Antiplatelet drug used- Aspirin	139 (with antiplatelet treatment- 12; without antiplatelet treatment- 127)	7
Sossdorf (2013) [38]	Germany	Retrospective cohort	Mean age of around 67 years Male (65%)	Timing of antiplatelet administration- after onset of sepsis Antiplatelet drug used- Aspirin	979 (data on nos. receiving and not receiving antiplatelet treatment not provided)	6
Otto (2013) [39]	Germany	Retrospective cohort	Mean age of around 66 years Male (60%)	Timing of antiplatelet administration- after onset of sepsis Antiplatelet drug used- Aspirin as well as clopidogrel	886 (with antiplatelet treatment- 187, aspirin; 8, clopidogrel; without antiplatelet treatment- 691)	8
Valerio-Rojas (2013) [40]	USA	Retrospective cohort	Median age of around 68 years Male (55%)	Timing of antiplatelet administration- after onset of sepsis Antiplatelet drug used- Aspirin (89%)	651 (with antiplatelet treatment- 272; without antiplatelet treatment- 379)	8
Losche (2012) [41]	Germany	Retrospective cohort	Mean age of around 65 years Male (57%)	Timing of antiplatelet administration- after onset of sepsis Antiplatelet drug used- Aspirin	834 (with antiplatelet treatment- 647; without antiplatelet treatment- 187)	7
Eisen (2012) [42]	Australia	Retrospective cohort	Mean age of around 65 years Male (67%)	Timing of antiplatelet administration- after onset of sepsis Antiplatelet drug used- Aspirin	970 (with antiplatelet treatment- 165; without antiplatelet treatment- 805)	8

* Wang et al. (2023) [29] analyzed two separate datasets and presented findings for each dataset individually, so these were considered as two distinct studies for analysis. “. The references for the two studies will be the same

Most (n=19) studies had a retrospective cohort design. The included studies were representative of varied geographical locations (Table 1). Notably, Wang et al. (2023) analyzed two separate datasets and presented findings for each dataset individually, so these were considered as two distinct studies for analysis [29]. In 13 studies, antiplatelet drugs were administered prior to the onset of sepsis and in 8 studies, they were administered after the onset of sepsis. Aspirin was the commonly used antiplatelet agent in most of the studies (n=18) (Table 1). Studies reported data on a total of 8,48,780 patients, with the mean NOS quality score of 7.38 out of the maximum attainable score of 9.

### Risk of mortality

For the risk of mortality analysis, the specific definitions for each time point of follow-up were as follows: *In-hospital mortality, i.e.*, mortality reported during the time of stay at the hospital; *mortality at 1 month*, i.e., mortality reported at any time from the time of discharge from the hospital until 30 days of discharge; *mortality at 3 months*, i.e., mortality reported at any time from the time of discharge from the hospital until 90 days of discharge, and, *mortality within 6–12 months of follow up*, i.e., mortality assessed at any time between 6–12 months of discharge from the hospital.

**Fig 2:**
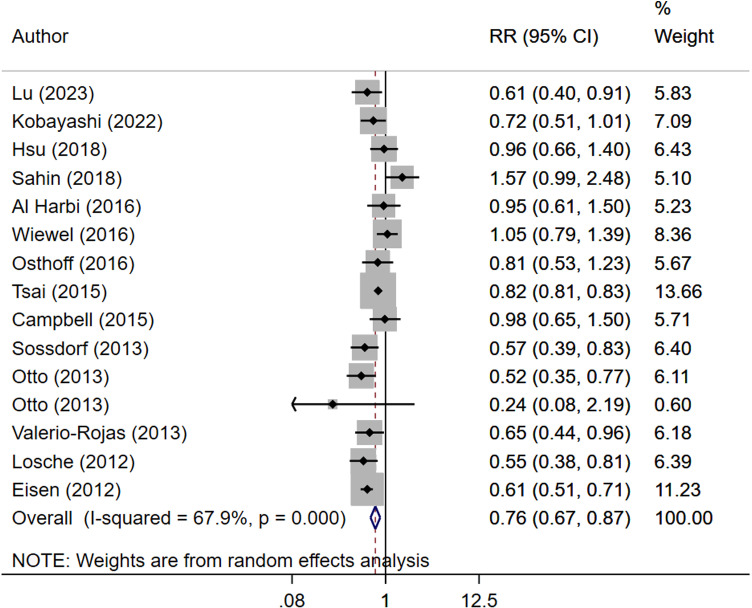
Risk of in-hospital mortality in septic patients with and without antiplatelet therapy (StataCorp. (2017). Stata version 15.0. StataCorp LP).

**Fig 3:**
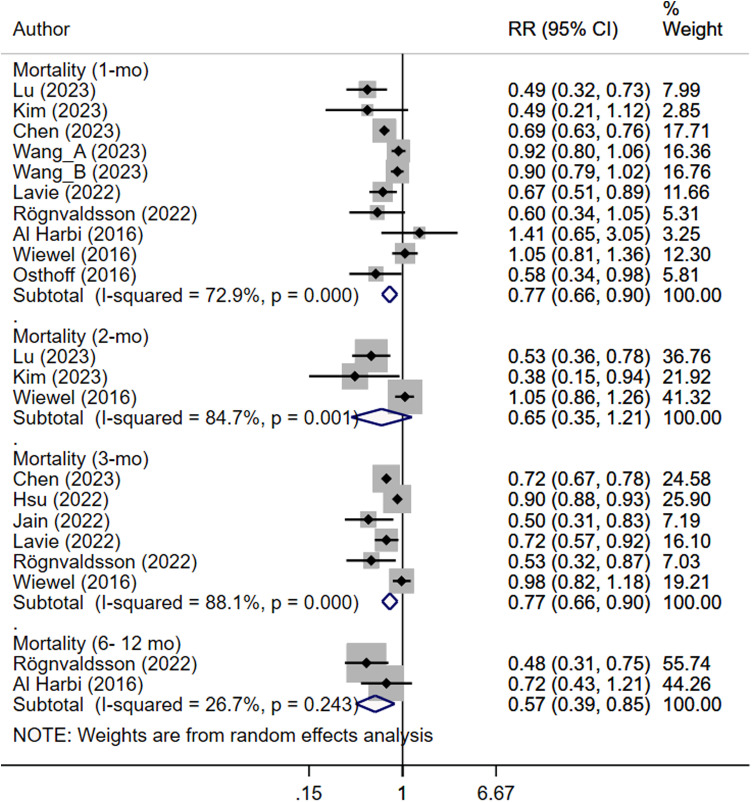
Risk of mortality at timepoints other than in-hospital mortality in septic patients with and without antiplatelet therapy (StataCorp. (2017). Stata version 15.0. StataCorp LP).

Septic patients that received antiplatelet therapy had significantly reduced risk of in-hospital mortality (RR 0.76, 95% CI: 0.67, 0.87; N=15, subjects=7,23,332; I^2^=67.9%), mortality at 1 month (RR 0.77, 95% CI: 0.66, 0.90; N=10, subjects=33,486; I^2^=72.9%), 3 months (RR 0.77, 95% CI: 0.66, 0.90; N=6, subjects= 1,07,023; I^2^=88.1%) and between 6–12 months of follow up (RR 0.57, 95% CI: 0.39, 0.85; N=2, subjects=1578; I^2^=26.7%) (Figs 2 and 3) compared to patients with sepsis who did not receive antiplatelet drugs, with no publication bias on Egger’s test (p=0.27 for in-hospital mortality; p=0.78 for mortality at 1-month follow up and p=0.18 for mortality at 3-month follow-up), and on funnel plots (S1–S4 Figs).

Subgroup analysis indicated that administration of anti-platelet drugs before the onset of sepsis reduced the risk of mortality at 1 month (RR 0.66, 95% CI: 0.50, 0.88; N=6, I^2^=63.2%) and 3 months of follow up (RR 0.79, 95% CI: 0.66, 0.94; N=5, I^2^=71.1%) but not the risk of in-hospital mortality (RR 0.88, 95% CI: 0.76, 1.03; N=8, I^2^=51.8%) (Table 2). On the other hand, administration of antiplatelet therapy after the onset of sepsis reduces the risk of in-hospital mortality (RR 0.61, 95% CI: 0.54, 0.69; N=7, I^2^=1.5%) but not the risk of mortality at 1-month follow up (RR 0.85, 95% CI: 0.70, 1.04; N=4, I^2^=83.9%) (Table 2). There was only one study examining the risk of mortality at 3-months of follow up with administration of anti-platelet drugs after onset of sepsis and therefore, conclusive evidence is lacking in this aspect (Table 2).

**Table 2:** Findings of the subgroup analysis.

	In-hospital mortality	Mortality at 1 month	Mortality at 3 months	Complications
	Pooled relative risk (RR) with 95% confidence intervals (N=number of studies; I^2^ represents degree of heterogeneity)			
**Timing of antiplatelet administration**				
Before sepsis onset	0.88 (0.76, 1.03) N=8; I^2^=51.8%	0.66 (0.50, 0.88)* N=6; I^2^=63.2%	0.79 (0.66, 0.94)* N=5; I^2^=71.1%	1.07 (0.85, 1.33) N=3; I^2^=0.0%
After sepsis onset	0.61 (0.54, 0.69)* N=7; I^2^=1.5%	0.85 (0.70, 1.04) N=4; I^2^=83.9%	0.72 (0.67, 0.78)* N=1	0.89 (0.58, 1.36) N=2; I^2^=46.7%
**Antiplatelet used**				
Aspirin	0.74 (0.61, 0.88)* N=11; I^2^=63.5%	0.74 (0.63, 0.86)* N=9; I^2^=71.5%	0.76 (0.63, 0.91)* N=4; I^2^=91.6%	1.01 (0.84, 1.21) N=5; I^2^=0.0%
Other (Clopidogrel/ Dipyridamole/ Prasugrel/ ticlopidine)	0.84 (0.70, 1.01) N=4; I^2^=46.6%	1.05 (0.81, 1.36) N=1	0.73 (0.38, 1.40) N=2; I^2^=84.2%	----
**Sample size**				
≥500	0.72 (0.63, 0.82)* N=13; I^2^=65.8%	0.78 (0.67, 0.91)* N=9; I^2^=75.0%	0.77 (0.66, 0.90)* N=6; I^2^=88.1%	0.96 (0.75, 1.24) N=4; I^2^=0.0%
<500	1.23 (0.77, 1.95) N=2; I^2^=54.8%	0.49 (0.21, 1.13) N=1	----	1.06 (0.82, 1.37) N=1
	**Length of ICU admission (days)**		**Length of hospital stay (days)**	
	Weighted mean difference (WMD) with 95% confidence intervals (N=number of studies; I^2^ represents degree of heterogeneity)			
**Timing of antiplatelet administration**				
Before sepsis onset	‒0.42 (‒1.29, 0.45) N=3; I^2^=97.7%		1.22 (‒1.51, 3.95) N=3; I^2^=93.3%	
After sepsis onset	‒0.05 (‒0.45, 0.34) N=4; I^2^=97.7%		0.25 (‒1.00, 1.50) N=3; I^2^=93.3%	
**Antiplatelet used**				
Aspirin	‒0.06 (‒0.34, 0.22) N=6; I^2^=96.5%		0.63 (‒0.66, 1.92) N=6; I^2^=93.2%	
Other (Clopidogrel/ Dipyridamole/ Prasugrel/ ticlopidine)	‒1.00 (‒1.17, ‒0.83) N=1; ---		---	
**Sample size**				
≥500	‒0.24 (‒0.54, 0.06) N=6; I^2^=97.6%		0.43 (‒0.87, 1.73) N=5; I^2^=94.3%	
<500	3.04 (‒1.41, 7.49) N=1; ---		5.40 (‒0.57, 11.4) N=1; ---	

*statistically significant at P<0.05

Use of aspirin as an antiplatelet drug was consistently linked to reduced risk of in-hospital mortality (RR 0.74, 95% CI: 0.61, 0.88; N=11, I^2^=63.5%) and mortality at 1 (RR 0.74, 95% CI: 0.63, 0.86; N=9, I^2^=71.5%) and 3 months (RR 0.76, 95% CI: 0.63, 0.91; N=4, I^2^=91.6%) of follow up (Table 2). Other antiplatelet drugs such as Clopidogrel/ Dipyridamole/Prasugrel/ ticlopidine did not correlate with lower risk of mortality at any of the timepoints of assessment. However, there were very few studies exploring these associations with non-aspirin drugs. Subgroup analysis based on the sample size indicated that in larger studies (i.e., 500 or more patients) the use of antiplatelet drugs was associated with reduced risk of mortality at hospital (RR 0.72, 95% CI: 0.63, 0.82; N=13, I^2^=65.8%), 1 month (RR 0.78, 95% CI: 0.67, 0.91; N=9, I^2^=75.0%) and 3 months (RR 0.77, 95% CI: 0.66, 0.90; N=6, I^2^=88.1%) of follow up (Table 2). On the other hand, pooling of studies with smaller (<500) sample size did show any significant association of use of antiplatelet drugs with reduced risk of mortality (Table 2).

### Risk of complications

The reported complications were gastrointestinal bleeding, thrombocytopenia, renal failure and respiratory failure. Lu et al. (2023) found that 38% of subjects receiving antiplatelet drugs experienced thrombocytopenia, compared to 36% in the control group [26]. Gastrointestinal bleeding occurred in approximately 5% of the antiplatelet group and 3% of the comparator group [26]. Kim et al. (2023) reported a combined incidence of complications (mainly septic shock, renal failure, and respiratory failure) at 54% in the antiplatelet group and 51% in the control group [27]. Chen et al. (2023) documented an acute renal failure incidence of 36.5% in the antiplatelet group and 37.0% in the control group, while gastrointestinal bleeding was reported at 0.7% and 1%, respectively [28]. Lavie et al. (2022) noted a 4% incidence of gastrointestinal bleeding in both groups [14]. Valerio-Rojas et al. (2013) observed an 80% incidence of acute renal injury in both groups [40].

**Fig 4:**
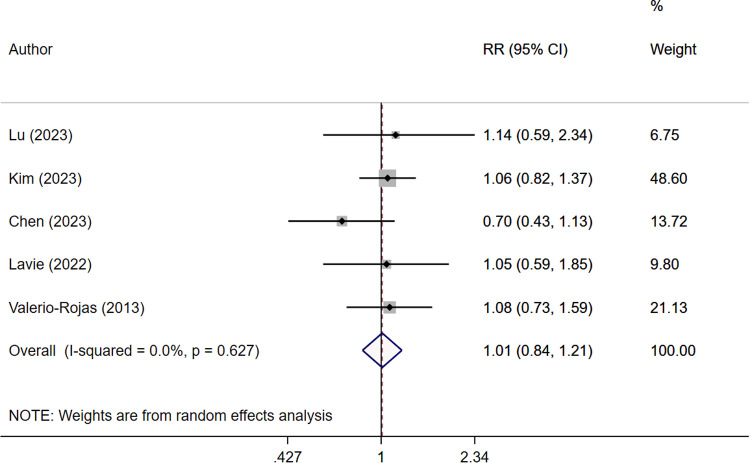
Risk of complications in septic patients with and without antiplatelet therapy (StataCorp. (2017). Stata version 15.0. StataCorp LP).

All groups of patients with sepsis had similar risk of complications (RR 1.01, 95% CI: 0.84, 1.21; N=5, I2=0.0%) (Fig 4), with no evidence of publication bias (S5 Fig). Subgroup analysis indicated that irrespective of the timing of administration of anti-platelet drugs, i.e., before (RR 1.07, 95% CI: 0.85, 1.33; N=3, I2=0.0%) or after (RR 0.89, 95% CI: 0.58, 1.36; N=2, I2=46.7%) the onset of sepsis, the risk of complications was comparable to that of patients who did not receive antiplatelet drugs (Table 2). Further, use of aspirin as an antiplatelet agent did not increase the risk of complications (RR 1.01, 95% CI: 0.84, 1.21; N=5, I^2^=0.0%) (Table 2). Subgroup analysis based on the sample size indicated that irrespective of the sample size, use of antiplatelet drugs did not correlate with increased risk of complications (Table 2).

### Duration of intensive care unit and hospital stay

**Fig 5:**
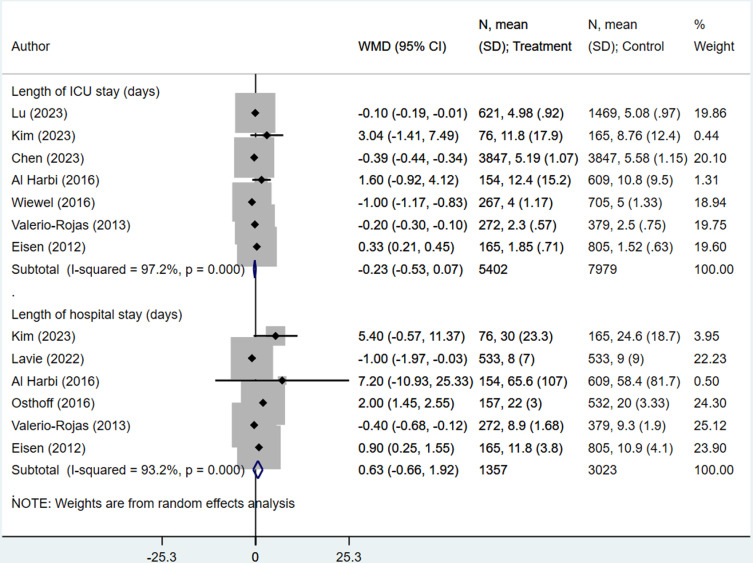
Length of intensive care unit (ICU) stay (in days) and overall hospital stay (in days) in septic patients with and without antiplatelet therapy (StataCorp. (2017). Stata version 15.0. StataCorp LP).

ICU stay (in days) (WMD -0.23, 95% CI: -0.53, 0.07; N=7, I^2^=97.2%) and overall hospital stay (in days) (WMD 0.63, 95% CI: -0.66, 1.92; N=6, I^2^=93.2%) were comparable in all groups (Fig 5). The results indicate that, on average, the ICU stay was 0.23 days shorter and duration of overall hospital stay was 0.63 day longer in the intervention group, compared to the control group, though the confidence interval includes zero, suggesting that the difference may not be statistically significant. Subgroup analysis did not show any difference in the length of ICU stay or overall hospital stay between the two groups, irrespective of the timing and type of antiplatelet therapy as well as the sample size (Table 2). The high heterogeneity persisted even in the subgroup analyses.

### Findings on GRADE certainty of evidence

The GRADE certainty of evidence for each of the outcomes is presented in Table 3. For the outcomes considered, the certainty varied from “low” to “very low” primarily because of inherent risk of bias, high heterogeneity and concerns of imprecision.

**Table 3:** Certainty of pooled evidence using the GRADE approach.

	Number of studies with design	Certainty of the evidence (GRADE)	Effect size (95% CI); I^2^	Reason for downgrading
**Risk of in-hospital mortality**	N=15 (All observational)	Low	RR 0.76 (95% CI: 0.67, 0.87); 67.9%	Risk of bias; high inconsistency
**Risk of mortality at 1 month**	N=10 (All observational)	Low	RR 0.77 (95% CI: 0.66, 0.90); 72.9%	Risk of bias; high inconsistency
**Risk of mortality at 3 months**	N=6 (All observational)	Low	RR 0.77 (95% CI: 0.66, 0.90); 88.1%	Risk of bias; high inconsistency
**Risk of mortality within 6–12 months of follow up**	N=2 (All observational)	Low	RR 0.57 (95% CI: 0.39, 0.85); 26.7%	Risk of bias; concerns of indirectness
**Risk of complications**	N=5 (All observational)	Low	RR 1.01 (95% CI: 0.84, 1.21); 0.0%	Risk of bias; concerns of imprecision
**Duration of Intensive care unit stay (days)**	N=7 (All observational)	Very Low	WMD ‒0.23 (95% CI: ‒0.53, 0.07); 97.2%	Risk of bias; concerns of imprecision; high inconsistency
**Duration of hospital stay**	N=6 (All observational)	Very Low	WMD 0.63 (95% CI: ‒0.66, 1.92); 93.2%	Risk of bias; concerns of imprecision; high inconsistency

## Discussion

This study highlights a significant reduction in mortality risk associated with antiplatelet therapy in patients with sepsis. Specifically, antiplatelet treatment markedly decreased in-hospital mortality and mortality at both 1-month and 3-month follow-ups. This consistent risk reduction across different time points underscores the potential benefit of antiplatelet therapy in improving survival outcomes in septic patients. Despite these positive findings, the therapy did not affect the duration of ICU or overall hospital stay, nor did it increase the risk of complications.

Our study confirms the results of the previous review by Ouyang et al that pooled data from 10 cohort studies and found a significant decrease in the mortality risk when antiplatelet drugs (OR=0.82), particularly aspirin, were used [19]. Similar to our subgroup analysis, this review also found that the use of antiplatelet drugs before (OR=0.78) or after sepsis onset (OR=0.59) can lower the mortality [19].

The observed reduction in mortality with antiplatelet therapy may stem from its multifaceted impact on sepsis pathophysiology. Antiplatelet drugs, such as aspirin, help mitigate microvascular thrombosis and improve tissue perfusion by inhibiting platelet aggregation [43–46]. This action supports endothelial function and prevents platelet-induced vascular damage. Additionally, these drugs enhance fibrinolysis, which is crucial in managing disseminated intravascular coagulation (DIC) often seen in sepsis [47–50]. Antiplatelet therapy also influences immune responses by modulating cytokine release and reducing systemic inflammation, partly by disrupting platelet-leukocyte aggregates [51–54]. Moreover, by decreasing oxidative stress linked to platelet activation, antiplatelet drugs may further protect against cellular damage, contributing to improved survival outcomes in septic patients [53,55].

Our findings particularly indicate that while aspirin use was consistently associated with reduced mortality in septic patients, other antiplatelet agents such as clopidogrel, dipyridamole, prasugrel, and ticlopidine did not yield significant survival benefits. This discrepancy may stem from key pharmacodynamic differences between these agents. Aspirin irreversibly inhibits cyclooxygenase-1 (COX-1), reducing thromboxane A2 synthesis, which not only suppresses platelet aggregation but also dampens inflammatory pathways involved in sepsis [56]. In contrast, P2Y12 inhibitors like clopidogrel act by blocking ADP-mediated platelet activation [57]. The available evidence demonstrates that clopidogrel is associated with significant variability in platelet response between patients, i.e., around 30% of patients undergoing treatment display high residual platelet reactivity [58,59]. Additionally, clopidogrel requires hepatic metabolism for activation, which may be impaired in critically ill patients with sepsis-related organ dysfunction [60]. Genetic variability in CYP2C19 metabolism could also contribute to inconsistent efficacy of P2Y12 inhibitors [61,62].

Beyond platelet inhibition, aspirin’s immunomodulatory effects may further explain its observed benefits. By inhibiting nuclear factor kappa B (NF-κB) signaling, aspirin reduces the production of pro-inflammatory cytokines such as TNF-α and IL-1, mitigating the excessive inflammatory response seen in sepsis [63]. In contrast, P2Y12 inhibitors primarily modulate platelet activity without these broader anti-inflammatory properties. The limited number of studies assessing non-aspirin agents also restrict definitive conclusions on their effectiveness.

The finding that antiplatelet drugs did not increase risk of complications underscores the potential safety profile of antiplatelet therapy in the context of sepsis management. Our findings suggest that these medications can be integrated into the treatment regimen without compromising patient safety. Nevertheless, it is crucial to interpret these results with a caution, considering the heterogeneity of sepsis presentations and the potential variations in patient characteristics across the included studies. In the context of the discussion around the safety of antiplatelet agents, it is worth mentioning the PLATO (Platelet Inhibition and Patient Outcomes) trial [64]. This was a large, multinational clinical trial that compared ticagrelor with clopidogrel in patients with acute coronary syndromes (ACS) [64]. The trial demonstrated that ticagrelor significantly reduced the rate of death from vascular causes, myocardial infarction, and stroke compared to clopidogrel, leading to its widespread adoption in clinical practice. However, controversy arose later regarding the trial’s findings, particularly concerning sepsis-related deaths. A disproportionate number of sepsis deaths were reported in the ticagrelor group compared to the clopidogrel group [18]. This raised concerns about whether ticagrelor might increase the risk of infection-related mortality. There were speculations that ticagrelor’s stronger antiplatelet effects could impair immune function or alter the host’s defence against infections, potentially leading to worse outcomes in sepsis [65]. The debate prompted further analyses of the data, but the exact reasons for the observed differences in sepsis deaths remained largely unclear. Despite the controversy, ticagrelor remains a key drug in managing ACS, but the discussion over its safety in the context of sepsis highlights the complexity of balancing antiplatelet therapy’s benefits and risks, particularly in vulnerable patient populations.

The evolving landscape of sepsis management, particularly after the COVID-19 pandemic, has influenced treatment approaches, including the potential role of antiplatelets. The hypercoagulable state observed in COVID-19-associated sepsis led to an increased focus on antithrombotic strategies, prompting multiple clinical trials evaluating the use of antiplatelets and anticoagulants in critically ill patients. Recent studies have explored the role of antiplatelet therapy in sepsis management, especially in the context of COVID-19. However, current guidelines have not incorporated antiplatelet agents into standard sepsis treatment protocols. Recent large-scale trials such as COVID-PACT and ACTIV-4a found that while full-dose anticoagulation reduced thrombotic complications, the addition of antiplatelets (e.g., clopidogrel) did not provide significant mortality benefits in COVID-19-associated sepsis [66,67]. Similarly, a systematic review and meta-analysis concluded that antiplatelet therapy did not significantly impact mortality rates in COVID-19 patients [68]. These findings suggest that while antithrombotic therapy remains a key consideration in critically ill patients, the use of anti-platelet agents in sepsis requires careful patient selection and further evaluation of specific agents, timing, and dosing strategies.

Current sepsis management guidelines have not formally integrated antiplatelet therapy, and its routine use remains unestablished. The post-pandemic period has emphasized the need for standardized protocols in sepsis management, particularly regarding thrombosis prevention, inflammation control, and endothelial protection. Future research should explore whether newer antiplatelet agents, with additional anti-inflammatory properties, could provide improved outcomes in sepsis compared to aspirin or P2Y12 inhibitors. Additionally, understanding how antiplatelet therapy interacts with evolving treatment paradigms, including immune-modulating therapies used in post-pandemic sepsis will be critical in shaping future recommendations.

It is important to note that the effect of antiplatelet therapy in sepsis might vary based on timing, platelet localization, and impact on inflammation and coagulation. Some studies suggest that pre-sepsis antiplatelet use could have mortality benefits, however, there are other studies that show no impact [16,36]. However, the potential for rebound platelet activation and thromboinflammation upon withdrawal has not been extensively studied. Additionally, platelet localization may affect therapeutic outcomes, as microvascular-sequestered platelets contribute to endothelial dysfunction and organ failure, yet many studies do not differentiate between systemic and localized platelet activity. While antiplatelets mitigate platelet-leukocyte interactions and microvascular thrombosis, their effect on pro-inflammatory cytokines remains inconsistent, suggesting their benefit may stem more from thromboinflammatory modulation than direct immune suppression. Patients with high bleeding risk (e.g., thrombocytopenia, DIC) are often excluded from previous trials, limiting the generalizability of findings. Balancing thromboinflammation control with bleeding risk remains a clinical challenge. Future trials should evaluate optimal therapy duration, effects of withdrawal in chronic users, and comparative efficacy of different agents, while incorporating biomarkers of platelet activation and endothelial injury to identify the most suitable patient subgroups.

The applicability of antiplatelet therapy in sepsis is not only influenced by its clinical efficacy but also by healthcare access disparities, particularly in low- and middle-income countries (LMICs). Sepsis management in these settings faces significant challenges, including limited access to intensive care units (ICUs), delayed diagnosis due to inadequate laboratory facilities, and restricted availability of essential medications. While antiplatelet drugs such as aspirin are widely available and inexpensive, more potent agents like ticagrelor and prasugrel are costly and less accessible in resource-limited settings. Furthermore, monitoring platelet function, coagulation status, and potential bleeding risks, which is crucial for safe antiplatelet use, may not be feasible in many LMICs due to a lack of point-of-care testing and trained personnel. To improve the feasibility and effectiveness of antiplatelet therapy in sepsis management across diverse healthcare settings, future research should explore context-specific strategies, including cost-effective dosing regimens, simplified monitoring approaches, and risk-adapted protocols tailored to resource-limited environments.

Our meta-analysis demonstrated a consistent association between antiplatelet therapy and reduced sepsis-related mortality; however, significant heterogeneity was observed. This variability likely stems from differences in sepsis definitions, with some studies using Sepsis-2 criteria and others employing Sepsis-3, which emphasizes organ dysfunction and may capture patients with higher baseline mortality risk. Patient comorbidities likely influenced the observed outcomes, as differences in baseline health conditions were evident between groups. Notably, patients receiving antiplatelet therapy, particularly aspirin, had a higher prevalence of cardiovascular disease, a condition for which aspirin is commonly prescribed. This could introduce a selection bias, where patients on antiplatelets may have received more intensive cardiovascular monitoring and management, potentially contributing to better overall outcomes independent of the antiplatelet effect in sepsis. Furthermore, individuals with cardiovascular diseases often have underlying pro-thrombotic and inflammatory conditions, which may make them more responsive to the anti-inflammatory and endothelial-protective effects of aspirin. Additionally, healthcare settings and treatment protocols varied and there were differences in antiplatelet use protocols, including dosing and concomitant anticoagulation. All of these may have further influenced the observed effects and impact the generalizability of the findings. The absence of randomized controlled trials also limits the robustness of our analysis, while variations in the quality of included studies, including sample size and methodology, may affect the reliability of our conclusions. To address these issues, future research should focus on well-designed prospective studies with standardized protocols to provide clearer insights into the role of antiplatelet therapy in sepsis. Another limitation of our meta-analysis is the conversion of odds ratios (OR) to relative risks (RR) for consistency across studies. While we used a well-established method to approximate RR, this approach assumes a stable baseline risk, which may not fully capture variations in study populations.

## Conclusion

Our results demonstrate that antiplatelet therapy is associated with a reduction in mortality risk in patients with sepsis without an increase in complications, suggesting a favourable risk-benefit profile. However, it is important to acknowledge several limitations, including variability in patient populations, antiplatelet drug regimens and dosages used, sample sizes, and other potential biases that may influence the findings. These factors should be considered when interpreting the results. To strengthen the validity of our conclusions, further research is needed to address these limitations. Specifically, well-designed randomized controlled trials (RCTs) should investigate optimal dosage, timing of administration, and the comparative effectiveness of different antiplatelet agents. Additionally, patient stratification based on sepsis severity, baseline coagulation status, and comorbidities would help identify subgroups most likely to benefit while minimizing bleeding risks. Standardized outcome measures, including mortality at multiple time points, organ dysfunction scores, and inflammatory biomarker changes, should be incorporated in future studies. Our study provides a valuable assessment of the benefits and safety of antiplatelet therapy in sepsis and suggests that this treatment could be a promising adjunct in sepsis management. Future studies should also explore which patient populations are most likely to benefit from antiplatelet therapy and consider potential modifications to current sepsis management strategies.

## Supporting information

S1 Table Search strategy used to identify potential studies for inclusion. (DOCX)

S2 Table The number of articles found on the search. (XLSX)

S3 Table Newcastle Ottawa scale (NOS) based quality assessment of included cohort studies. (DOCX)

S4 Table PRISMA 2020 checklist. (DOCX)

S1 Fig Funnel plot for publication bias for risk of in-hospital mortality (StataCorp. (2017). Stata version 15.0. StataCorp LP).
(TIF)

S2 Fig Funnel plot for publication bias for risk of mortality at 1 month follow up (StataCorp. (2017). Stata version 15.0. StataCorp LP).
(TIF)

S3 Fig Funnel plot for publication bias for risk of mortality at 3 months follow up (StataCorp. (2017). Stata version 15.0. StataCorp LP).
(TIF)

S4 Fig Funnel plot for publication bias for risk of mortality between 6–12 months of follow up (StataCorp. (2017). Stata version 15.0. StataCorp LP).
(TIF)

S5 Fig Funnel plot for publication bias for risk of complications (StataCorp. (2017). Stata version 15.0. StataCorp LP).
(TIF)
