# Supplementary material for: Impact of antiplatelet therapy on outcomes of sepsis: A systematic review and meta-analysis
Source: PLoS One. 2025 Apr 29;20(4):e0322293. doi: 10.1371/journal.pone.0322293 (PMC12040142; doi:10.1371/journal.pone.0322293)
Supplement: S1 Table — (DOCX) [file pone.0322293.s001.docx]

**S1 Table. Search strategy used to identify potential studies for inclusion**

| **Search strategy in PubMed**  (("Antiplatelet Therapy"[MeSH] OR "Antiplatelet Agents"[MeSH] OR "Platelet Aggregation Inhibitors"[MeSH] OR “Fibrinolytic Agents” [MeSH] OR Aspirin OR Acetylsalicylic Acid OR Clopidogrel OR Dipyridamole OR Prasugrel OR Ticlopidine) AND ("Sepsis"[MeSH] OR "Severe Sepsis"[MeSH] OR "Septic Shock"[MeSH] OR "Septicaemia"[MeSH] OR "Systemic Inflammatory Response Syndrome"[MeSH] OR "SIRS"[All Fields]) AND ("Outcomes"[All Fields] OR "Mortality"[All Fields] OR "Survival"[All Fields] OR "Death"[MeSH] OR "Complications"[All Fields] OR "Adverse Outcomes"[All Fields]) AND (("Clinical Trial"[pt] OR "Randomized Controlled Trial"[pt] OR "Observational Study"[pt])) AND (english[lang]) AND ("0001/01/01"[PDAT] : "2023/11/01"[PDAT]) AND (Humans[MeSH])  **Search strategy in EMBASE**  ('antiplatelet therapy'/exp OR 'antiplatelet agent'/exp OR 'platelet aggregation inhibitor'/exp OR 'fibrinolytic agent'/exp OR 'aspirin'/exp OR 'acetylsalicylic acid'/exp OR 'clopidogrel'/exp OR 'dipyridamole'/exp OR 'prasugrel'/exp OR 'ticlopidine'/exp) AND ('sepsis'/exp OR 'severe sepsis'/exp OR 'septic shock'/exp OR 'septicaemia'/exp OR 'systemic inflammatory response syndrome'/exp OR 'SIRS') AND ('outcome assessment'/exp OR mortality OR survival OR 'death'/exp OR complications OR 'adverse outcome') AND ('clinical trial'/exp OR 'randomized controlled trial'/exp OR 'observational study'/exp) AND [english]/lim AND [humans]/lim AND [0001-01-01]/py TO [2023-11-01]/py  **Search strategy in Scopus**  TITLE-ABS-KEY ("Antiplatelet Therapy" OR "Antiplatelet Agents" OR "Platelet Aggregation Inhibitors" OR "Fibrinolytic Agents" OR Aspirin OR "Acetylsalicylic Acid" OR Clopidogrel OR Dipyridamole OR Prasugrel OR Ticlopidine) AND TITLE-ABS-KEY ("Sepsis" OR "Severe Sepsis" OR "Septic Shock" OR "Septicaemia" OR "Systemic Inflammatory Response Syndrome" OR SIRS) AND TITLE-ABS-KEY ("Outcomes" OR Mortality OR Survival OR Death OR Complications OR "Adverse Outcomes") AND (LIMIT-TO (DOCTYPE, "ar") OR LIMIT-TO (DOCTYPE, "re") OR LIMIT-TO (DOCTYPE, "cp")) AND (LIMIT-TO (LANGUAGE, "English")) AND (PUBYEAR < 2024 AND PUBYEAR >= 0001) |
| --- |
