# Supplementary material for: Impact of antiplatelet therapy on outcomes of sepsis: A systematic review and meta-analysis
Source: PLoS One. 2025 Apr 29;20(4):e0322293. doi: 10.1371/journal.pone.0322293 (PMC12040142; doi:10.1371/journal.pone.0322293)
Supplement: S3 Table — (DOCX) [file pone.0322293.s003.docx]

**S3 Table. Newcastle Ottawa scale (NOS) based quality assessment of included cohort studies**

|  | Lu (2023) | Kim (2023) | Chen (2023) | Wang_A (2023) | Wang_B (2023) | Kobayashi (2022) | Hsu (2022) | Jain (2022) | Lavie (2022) |
| --- | --- | --- | --- | --- | --- | --- | --- | --- | --- |
| 1. Representativeness of the exposed cohort  a) Truly representative  b) Somewhat representative  c) Representative of selected group of users  d) No description of the derivation of the cohort | 1 | 1 | 1 | 1 | 1 | 1 | 1 | 1 | 1 |
| 2. Selection of the non-exposed cohort  a) Drawn from the same community as the exposed cohort  b) Drawn from a different source  c) No description of the derivation of the non-exposed cohort | 1 | 1 | 1 | 1 | 1 | 1 | 1 | 1 | 1 |
| 3. Ascertainment of exposure  a) Secure record  b) Structured interview  c) Written self-report  d) No description | 1 | 1 | 1 | 1 | 1 | 1 | 1 | 1 | 1 |
| 4. Demonstration that outcome of interest was not present at start of study  a) Yes  b) No | 0 | 1 | 1 | 1 | 1 | 1 | 1 | 1 | 1 |
| 5. Comparability of cohorts on the basis of the design or analysis  a) Study controls for most important factors  b) Study controls for any additional factor | 1 | 1 | 1 | 1 | 1 | 1 | 1 | 0 | 1 |
| 6. Assessment of outcome  a) Independent blind assessment  b) Record linkage  c) Self report  d) No description | 1 | 1 | 1 | 1 | 1 | 1 | 1 | 1 | 1 |
| 7. Was follow-up long enough for outcomes to occur  a) Yes  b) No | 1 | 0 | 1 | 1 | 1 | 1 | 1 | 1 | 1 |
| 8. Adequacy of follow up of cohorts  a) Complete follow up - all subjects accounted for  b) Subjects lost to follow up unlikely to introduce bias or description provided of those lost)  c) Low follow up rate and no description of those lost  d) No statement reported | 1 | 1 | 1 | 1 | 1 | 1 | 1 | 1 | 1 |
| Total score | 7 | 7 | 8 | 8 | 8 | 8 | 8 | 7 | 8 |
| Note: A yes for an “underscored item” will lead to one point; however, a study can be awarded a maximum of one point for items numbered 1,2,3,4,6,7,8 and a maximum of two points can be given for items numbered 5 | | | | | | | | |  |

**Supplementary table (continued). Newcastle Ottawa scale (NOS) based quality assessment of included cohort studies**

|  | Rögnvaldsson (2022) | Hsu (2018) | Sahin (2018) | Wiewel (2016) | Osthoff (2016) | Tsai (2015) | Campbell (2015) | Sossdorf (2013) | Otto (2013) |
| --- | --- | --- | --- | --- | --- | --- | --- | --- | --- |
| 1. Representativeness of the exposed cohort  a) Truly representative  b) Somewhat representative  c) Representative of selected group of users  d) No description of the derivation of the cohort | 1 | 1 | 1 | 1 | 1 | 1 | 1 | 1 | 1 |
| 2. Selection of the non-exposed cohort  a) Drawn from the same community as the exposed cohort  b) Drawn from a different source  c) No description of the derivation of the non-exposed cohort | 1 | 0 | 1 | 1 | 1 | 1 | 1 | 1 | 1 |
| 3. Ascertainment of exposure  a) Secure record  b) Structured interview  c) Written self-report  d) No description | 1 | 1 | 0 | 1 | 1 | 1 | 1 | 1 | 1 |
| 4. Demonstration that outcome of interest was not present at start of study  a) Yes  b) No | 0 | 1 | 1 | 1 | 0 | 0 | 0 | 1 | 1 |
| 5. Comparability of cohorts on the basis of the design or analysis  a) Study controls for most important factors  b) Study controls for any additional factor | 2 | 1 | 0 | 1 | 1 | 1 | 1 | 0 | 1 |
| 6. Assessment of outcome  a) Independent blind assessment  b) Record linkage  c) Self report  d) No description | 1 | 1 | 1 | 1 | 1 | 1 | 1 | 1 | 1 |
| 7. Was follow-up long enough for outcomes to occur  a) Yes  b) No | 1 | 1 | 1 | 1 | 1 | 1 | 1 | 0 | 1 |
| 8. Adequacy of follow up of cohorts  a) Complete follow up - all subjects accounted for  b) Subjects lost to follow up unlikely to introduce bias or description provided of those lost)  c) Low follow up rate and no description of those lost  d) No statement reported | 1 | 1 | 1 | 1 | 1 | 1 | 1 | 1 | 1 |
| Total score | 8 | 7 | 6 | 8 | 7 | 7 | 7 | 6 | 8 |
| Note: A yes for an “underscored item” will lead to one point; however, a study can be awarded a maximum of one point for items numbered 1,2,3,4,6,7,8 and a maximum of two points can be given for items numbered 5 | | | | | | | | |  |

**S3 Table (continued). Newcastle Ottawa scale (NOS) based quality assessment of included cohort studies**

|  | Valerio-Rojas (2013) | Losche (2012) | Eisen (2012) |
| --- | --- | --- | --- |
| 1. Representativeness of the exposed cohort  a) Truly representative  b) Somewhat representative  c) Representative of selected group of users  d) No description of the derivation of the cohort | 1 | 1 | 1 |
| 2. Selection of the non-exposed cohort  a) Drawn from the same community as the exposed cohort  b) Drawn from a different source  c) No description of the derivation of the non-exposed cohort | 1 | 1 | 1 |
| 3. Ascertainment of exposure  a) Secure record  b) Structured interview  c) Written self-report  d) No description | 1 | 1 | 1 |
| 4. Demonstration that outcome of interest was not present at start of study  a) Yes  b) No | 1 | 1 | 1 |
| 5. Comparability of cohorts on the basis of the design or analysis  a) Study controls for most important factors  b) Study controls for any additional factor | 1 | 1 | 1 |
| 6. Assessment of outcome  a) Independent blind assessment  b) Record linkage  c) Self report  d) No description | 1 | 1 | 1 |
| 7. Was follow-up long enough for outcomes to occur  a) Yes  b) No | 1 | 0 | 1 |
| 8. Adequacy of follow up of cohorts  a) Complete follow up - all subjects accounted for  b) Subjects lost to follow up unlikely to introduce bias or description provided of those lost)  c) Low follow up rate and no description of those lost  d) No statement reported | 1 | 1 | 1 |
| Total score | 8 | 7 | 8 |
| Note: A yes for an “underscored item” will lead to one point; however, a study can be awarded a maximum of one point for items numbered 1,2,3,4,6,7,8 and a maximum of two points can be given for items numbered 5 | | | |

**S3 Table (continued). Newcastle Ottawa scale (NOS) based quality assessment of included case-control study**

|  | Al Harbi (2016) |
| --- | --- |
| 1. Is the case definition adequate?   a) Yes, with independent validation  b) Yes, eg record linkage or based on self reports c) no description | 1 |
| 1. Representativeness of the cases   a) Consecutive or obviously representative series of cases  b) Potential for selection biases or not stated | 1 |
| 1. Selection of Controls   a) Community controls  b) Hospital controls  c) No description | 1 |
| 4. Definition of Controls  a) No history of disease (endpoint)  b) No description of source | 1 |
| 5. Comparability of cases and controls on the basis of the design or analysis  a) Study controls for most important factors  b) Study controls for any additional factors | 1 |
| 6. Ascertainment of exposure  a) Secure record  b) Structured interview where blind to case/control status  c) Interview not blinded to case/control status  d) Written self report or medical record only  e) No description | 1 |
| 7. Same method of ascertainment for cases and controls  a) Yes  b) No | 1 |
| 8. Non-Response rate  a) Same rate for both groups  b) Non respondents described  c) Rate different and no designation | 0 |
| Total score | 7 |
| Note: A yes for an “underscored item” will lead to one point; however, a study can be awarded a maximum of one point for items numbered 1,2,3,4,6,7,8 and a maximum of two points can be given for items numbered 5 | |
