# Supplementary figures and images for: Impact of antiplatelet therapy on outcomes of sepsis: A systematic review and meta-analysis
Source: PLoS One. 2025 Apr 29;20(4):e0322293. doi: 10.1371/journal.pone.0322293 (PMC12040142; doi:10.1371/journal.pone.0322293)

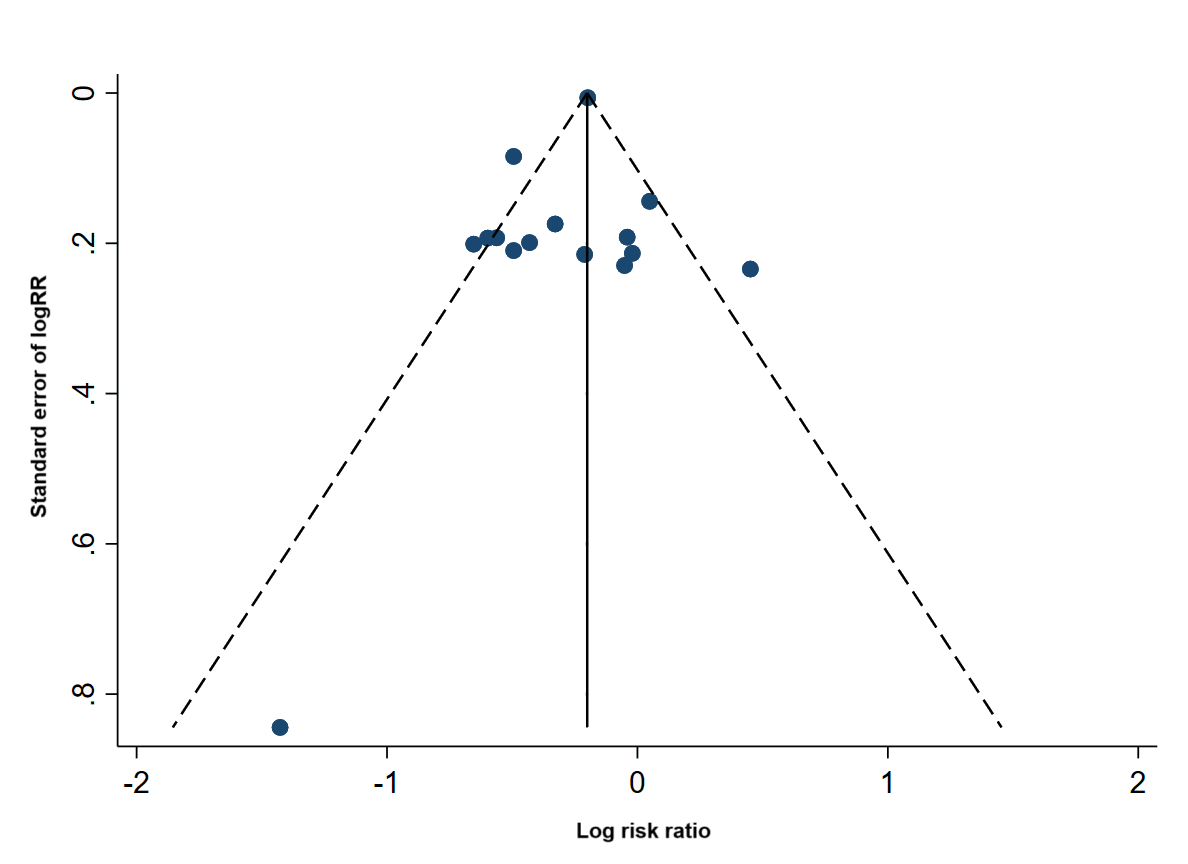

Supplement: S1 Fig — Stata version 15.0. StataCorp LP). (TIF) [file pone.0322293.s004.tif]

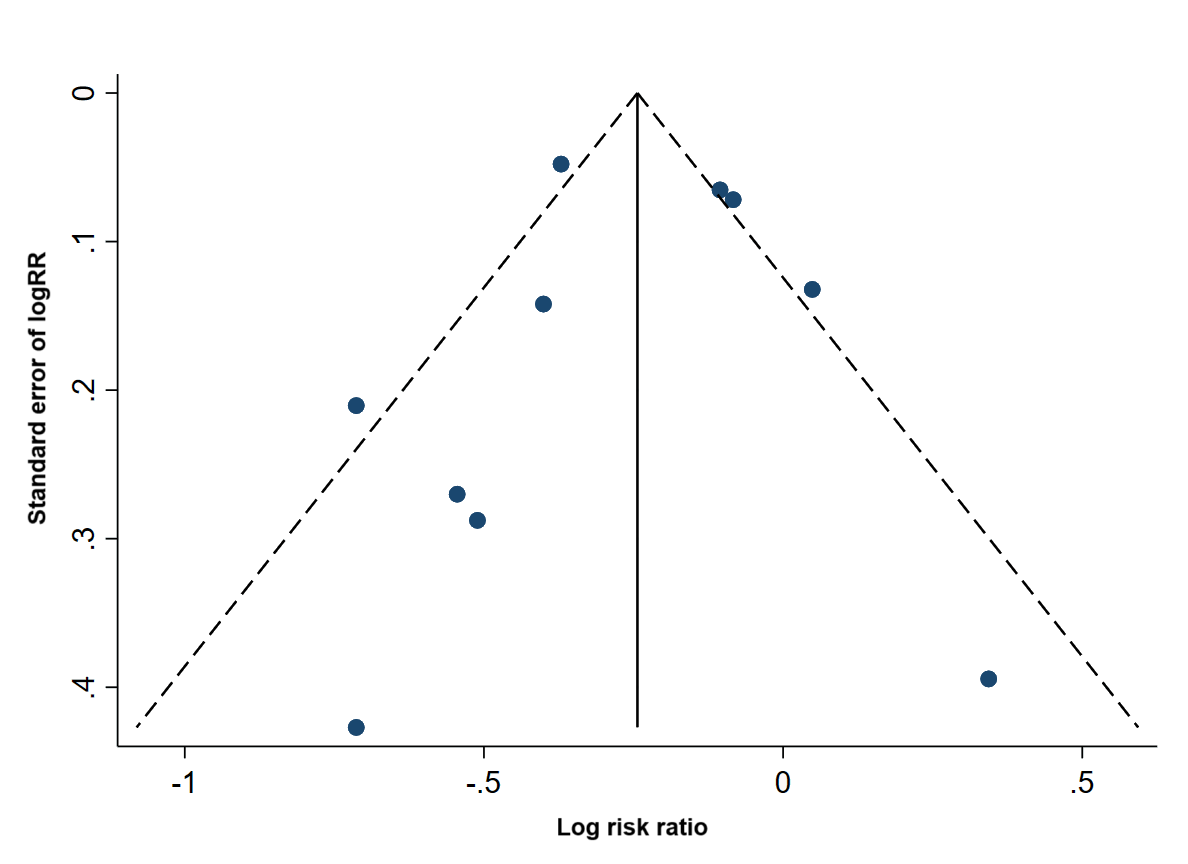

Supplement: S2 Fig — Stata version 15.0. StataCorp LP). (TIF) [file pone.0322293.s005.tif]

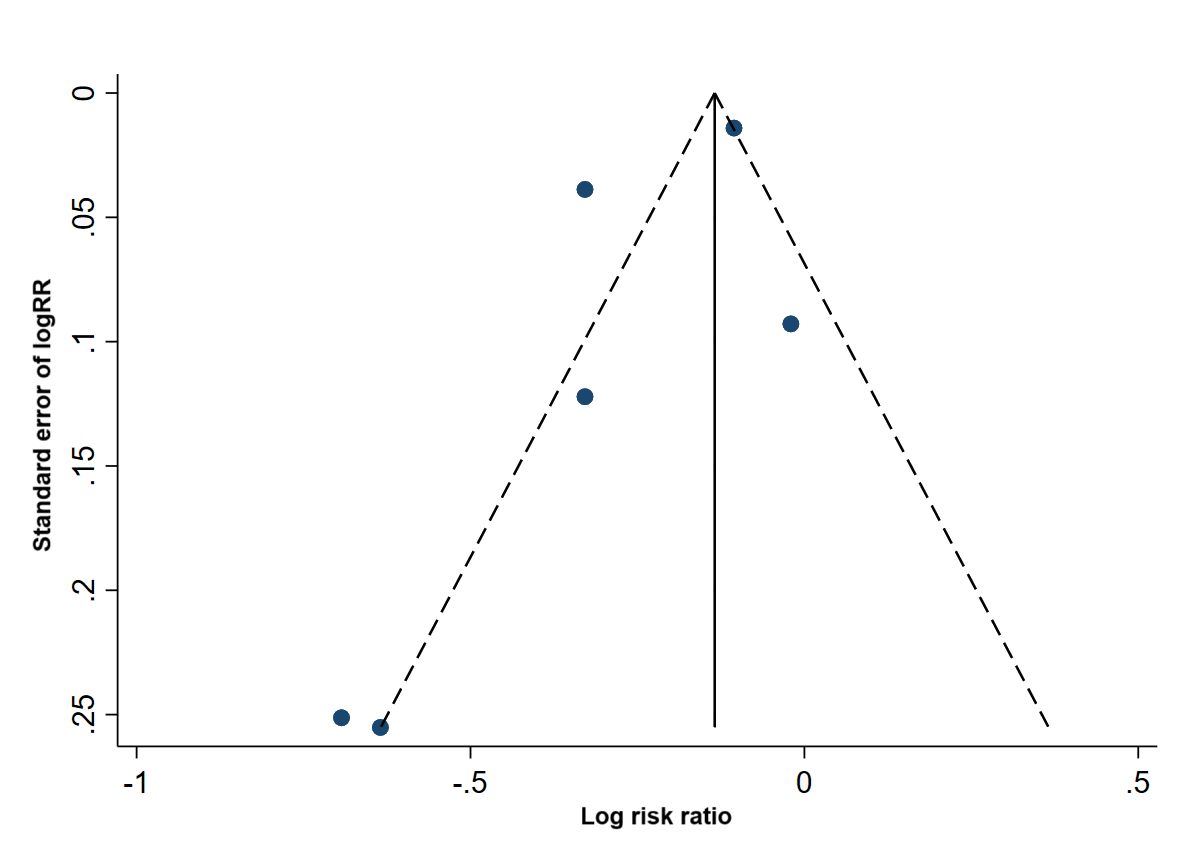

Supplement: S3 Fig — Stata version 15.0. StataCorp LP). (TIF) [file pone.0322293.s006.tif]

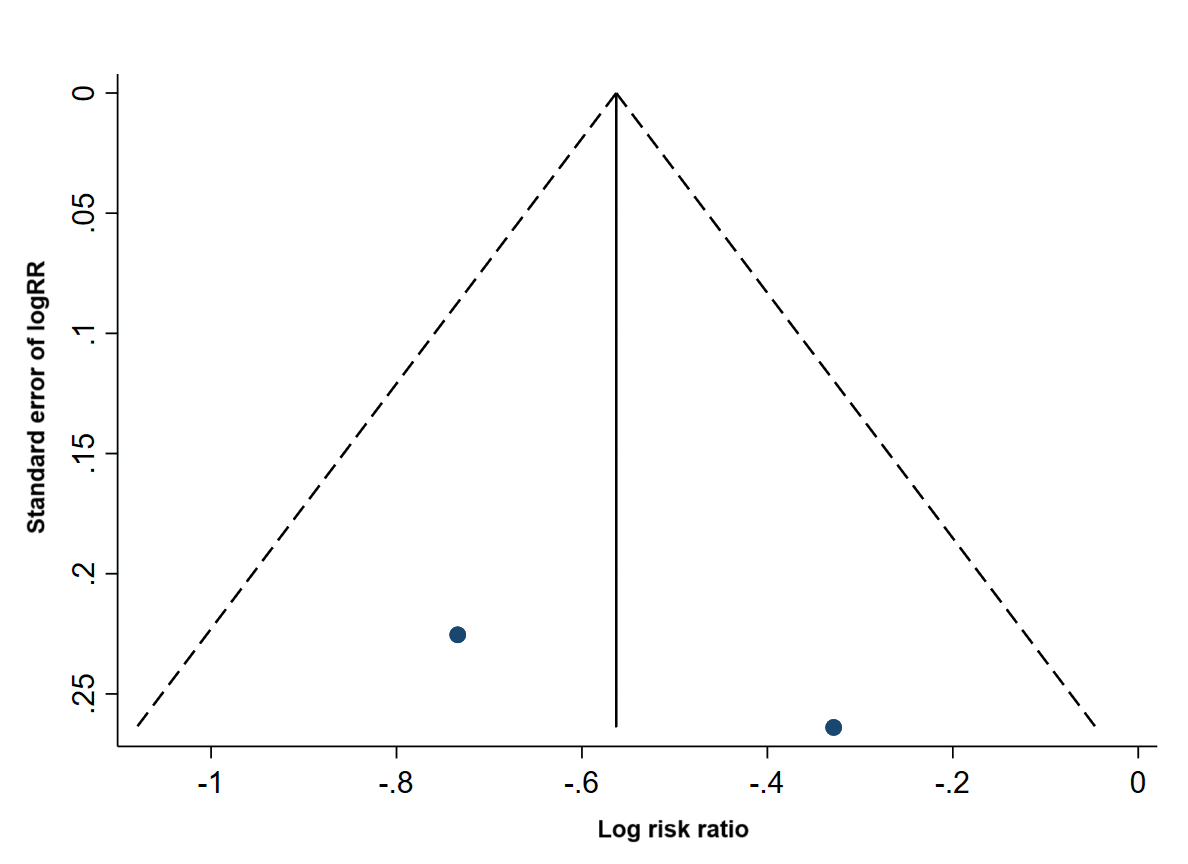

Supplement: S4 Fig — Stata version 15.0. StataCorp LP). (TIF) [file pone.0322293.s007.tif]

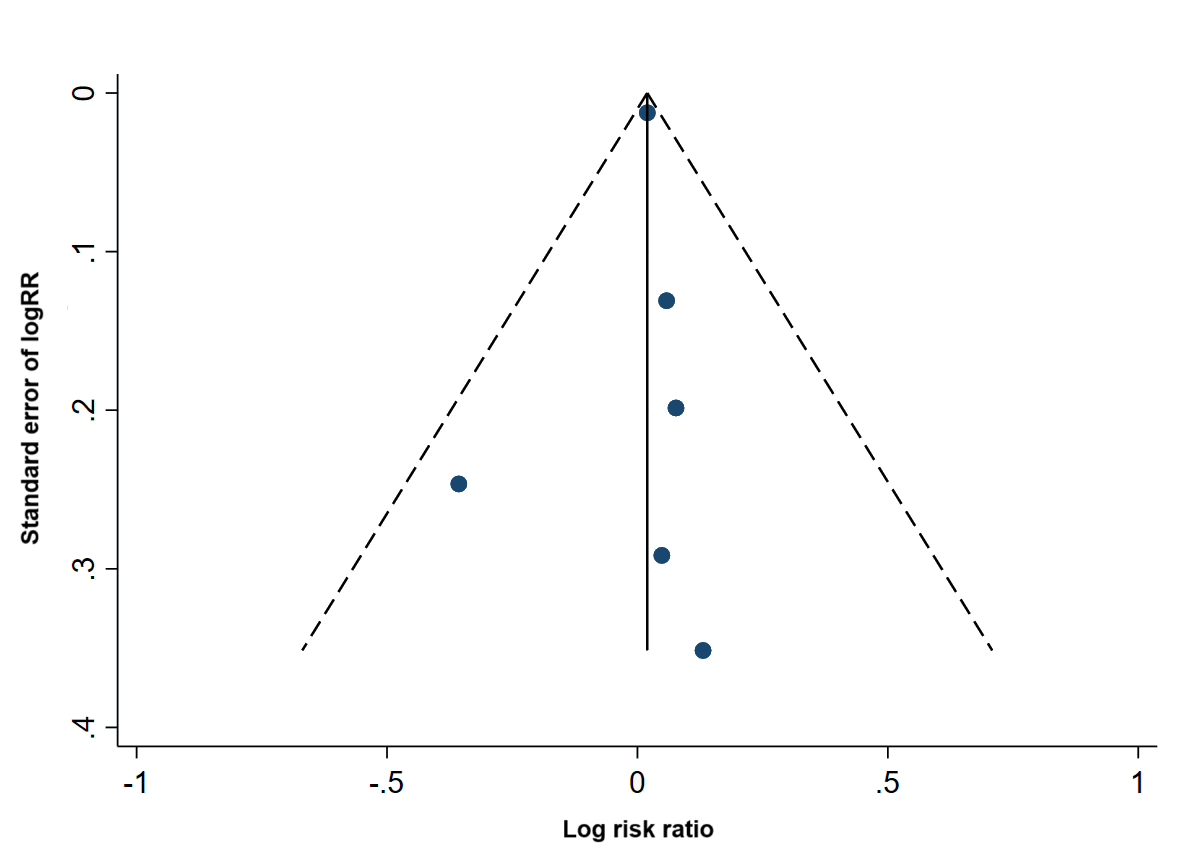

Supplement: S5 Fig — Stata version 15.0. StataCorp LP). (TIF) [file pone.0322293.s008.tif]
